# Lateral Laxity in Flexion Influences Patient-Reported Outcome After Total Knee Arthroplasty

**DOI:** 10.1007/s43465-023-01045-8

**Published:** 2023-12-07

**Authors:** Nobukazu Okamoto, Eiichi Nakamura, Tetsuro Masuda, Satoshi Hisanaga, Takeshi Miyamoto

**Affiliations:** 1 Department of Orthopaedic Surgery, Faculty of Life Sciences, Kumamoto University Hospital, Kumamoto University, 1-1-1 Honjo, Kumamoto, Japan; 2 Department of Orthopaedic Surgery, Kumamoto Kaiseikai Hospital, Kumamoto University, Kumamoto, Japan

**Keywords:** Total knee arthroplasty, Total knee replacement, Lateral laxity, Patient-reported outcome, Satisfaction

## Abstract

**Introduction:**

Slight lateral laxity exists in normal knee especially in flexion. The lateral laxity in flexion has possibility to affect the outcome after total knee arthroplasty (TKA).

**Purpose:**

The purpose of this study was to determine how intraoperative laxity in flexion affects patient-reported outcome after total knee arthroplasty.

**Methods:**

We retrospectively analysed 98 knees with osteoarthritis that underwent total knee arthroplasty. After bone resection, ligament imbalance and joint component gaps were measured using an offset-type tensor while applying a 40-lb joint distraction force at 0° and 90° of knee flexion. The lateral laxity in flexion was determined by subtracting polyethylene insert thickness from the lateral gap at 90°. All patients were divided into three groups: ≤ 2 mm (A), 2–5 mm (B), and > 5 mm (C). One year after surgery, patients were asked to fill out questionnaires using the new Knee Society Score after examination outside the consultation room.

**Results:**

The mean intraoperative lateral laxities at 90° were − 0.2 ± 2.1 mm, 3.5 ± 0.7 mm, and 6.7 ± 1.9 mm in groups A, B, and C, respectively. The symptom score of group C was significantly lower than those of groups A or B. There were no significant differences in terms of satisfaction or the expectation and activity scores among all groups. There were no significant differences in terms of alignment after total knee arthroplasty among all groups.

**Conclusions:**

Excessive lateral laxity possibly resulted in worse patient-reported outcomes. However, slight lateral laxity of 2–5 mm might have no effect on patient-reported outcome and this slight varus imbalance could be acceptable. Altogether, our findings would lead to avoidance of excessive medial release in soft tissue balancing.

## Introduction

Total knee arthroplasty (TKA) is a procedure designed to provide pain relief for patients with advanced osteoarthritis and to improve their quality of life over the long term. However, satisfaction and/or expectations after TKA were lower than those after total hip arthroplasty (THA) [1, 2]. Patient-reported outcome has been recognised as an important evaluation after TKA. Recently, in 2011, the Knee Society Scoring System (2011 KSS) was developed to evaluate patient satisfaction, expectations and physical activities and has since been widely used [3]. Factors affecting patient satisfaction have been reported to be postoperative range of motion (ROM) as well as coronal and rotational alignment [4, 5].

Traditionally, balancing TKA should occur in a rectangular gap, equal in both extension and flexion. Previously, it has been reported that excessive lateral laxity would cause worse knee function [6]. However, to achieve this balance, surgeons often need to release medial soft tissue, for example the medial collateral ligament and semimembranosus tendon. This might cause medial instability and knee dysfunction. Medial laxity more than 3 or 4 mm in flexion after TKA has been reported to the cause of low subjective outcomes [7–10]. Moreover, although the normal knee has slight lateral laxity, especially in flexion, few studies have reported the lateral laxity in flexion affect the outcomes after total knee arthroplasty (TKA) [11, 12].

The purpose of this study was to determine how the intraoperative laxity in flexion affects patient-reported outcomes (2011 KSS) after TKA. Hypothesis was that slight lateral laxity similar to that of the normal knee improved post-operative patient-reported outcome.

## Methods

We retrospectively analysed 98 knees in patients with medial osteoarthritis excluding the rheumatoid arthritis patients who underwent TKA using fixed-bearing TKA (NexGen LPS Flex, Zimmer). All patients provided informed consent to participate in this institutional review board-approved study.

### Surgical Technique and Measurements

**Fig. 1:**
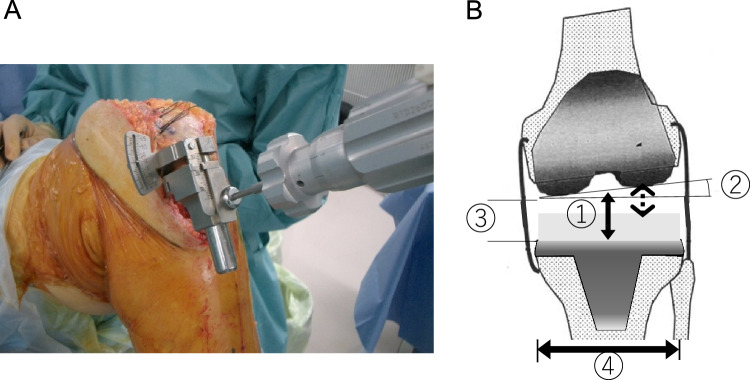
Measurement of the lateral laxity. **A** Ligament imbalance and joint component gap were measured using an offset-type tensor while applying 40-lb joint distraction force at 0° and 90° of knee flexion. **B** The lateral laxity in flexion (L) (dotted arrow) was determined as the value obtained after subtracting polyethylene insert thickness (1) from the lateral centre gap at 90° calculated using the ligament imbalance, (2) joint component gap, (3) and tibial component width (4)

The mean age at surgery was 78 years. All TKAs were performed using the measured resection technique. The knee incision was made through a medial parapatellar approach. The patella was not resurfaced. The distal femur was cut perpendicularly to the femoral mechanical axis. Femoral rotation was set parallel to the surgical epicondylar axis using epicondylar view radiography. Femoral rotations calculated preoperatively were 3° or 5° of external rotation from the posterior condylar axis. The proximal tibia was cut perpendicularly to the tibial mechanical axis. Tibial rotation was set to Akagi’s line. The ligament balance was adjusted to less than 2 mm in extension using the stepped medial release and spacer block method. After bone resection, ligament imbalance and joint component gap were measured using an offset-type tensor while applying a 40-lb joint distraction force at 0° and 90° of knee flexion (Fig. 1A) [13]. The lateral laxity (L) in flexion was determined as the value obtained after subtracting polyethylene insert thickness from the lateral centre gap at 90°, calculated using the ligament imbalance, joint component gap, and tibial component width (Fig. 1B). All patients were divided into three groups based on the measure of lateral laxity in flexion: ≤ 2 mm (A), 2–5 mm (B), and > 5 mm (C). At the 1-year follow-up after the surgery radiographic evaluation was performed using full leg-length standing radiographs to obtain the hip–knee–ankle (HKA) angle and the patients were asked to fill the questionnaires by themselves using 2011 KSS after examination outside the consultation room.

### Statistical Analysis

Spearman correlation coefficients were used to assess the associations between lateral laxity and KSS scores. The Kruskal–Wallis test was used for comparisons among the three groups. Pair-wise comparisons with Bonferroni correction were used to determine the differences between the groups. P-values less than 0.05 were considered significant.

## Results

**Fig. 2:**
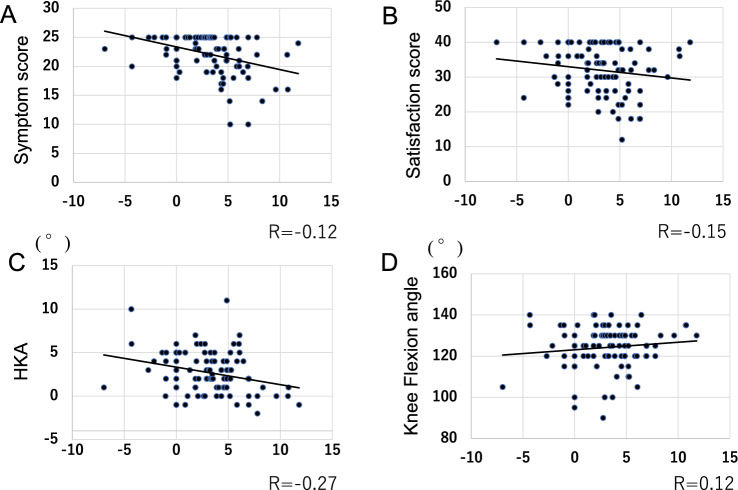
The scatter plot of correlation between lateral laxity in flexion (L) and clinical scores or alignment. **A** Symptom score, **B** satisfaction score, **C** hip–knee angle, and **D** knee flexion angle

**Fig. 3:**
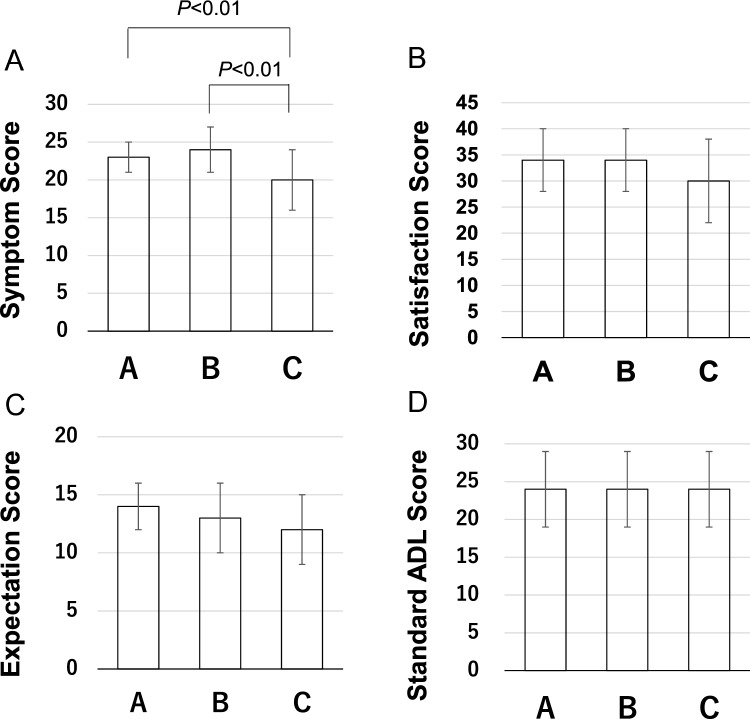
The graph of the Knee Society Score among three groups. **A** Symptom score, **B** satisfaction score, **C** expectation score, and **D** standard activities of daily living score

Lateral laxity in flexion (L) was not significantly correlated with the elements of the 2011 KSS scores or postoperative ROM (Fig. 2A–C). However, HKA angle negatively correlated with lateral laxity in flexion (Fig. 2D). After the patients were divided into three groups, there were 34 patients in group A, 34 in group B, and 30 in group C. Preoperative data including ROM were not significantly different among groups (Table 1). The flexion gap was larger in all groups than extension gap (Table 2). The varus imbalances in extension and flexion were significantly different between each group (Table 2). The mean intraoperative lateral laxity of group B and C was larger than that of group A (Table 2). There were no significant differences among the three groups in terms of alignment and postoperative ROM (Table 3). The symptom score of group C was significantly lower than those of groups A or B (Fig. 3A). Scores in group A were not significantly different from those of group B (Fig. 3A). There were no significant differences in terms of satisfaction or expectation scores or in terms of activity scores among all groups (Fig. 3B–D).

**Table 1:** Preoperative demographic data

	Group A (*n* = 34)	Group B (*n* = 34)	Group C (*n* = 30)
Patient age (years)	76 ± 6	77 ± 5	76 ± 7
Sex (female/male)	30/4	27/7	24/6
Knee extension (°)	7 ± 8	7 ± 6	7 ± 7
Knee flexion (°)	121 ± 14	124 ± 14	122 ± 14

Values are expressed as mean ± SD (range)

**Table 2:** Intraoperative balancing data

	Group A (*n* = 34)	Group B (*n* = 34)	Group C (*n* = 30)
Gap difference (flex-ext) (mm)	2.5 ± 1.8	2.5 ± 1.7	3.6 ± 2.8
Varus imbalances in extension (°)	0.5 ± 1.2	1.4 ± 2.6	2.7 ± 1.8
Varus imbalances in flexion (°)	0.1 ± 2.4	2.8 ± 2.6	4.9 ± 2.3
Lateral laxity in extension (mm)	0.9 ± 2.5	3.1 ± 2.0	2.8 ± 2.7
Lateral laxity in flexion (L) (mm)	− 0.2 ± 2.1	3.5 ± 0.7	6.7 ± 1.9

Values are expressed as mean ± SD (range)

**Table 3:** Postoperative data

	Group A (*n* = 34)	Group B (*n* = 34)	Group C (*n* = 30)
HKA angle (°)	183 ± 3	183 ± 2	182 ± 3
Knee extension (°)	1 ± 3	1 ± 3	2 ± 3
Knee flexion (°)	124 ± 11	124 ± 11	125 ± 8

Values are expressed as mean ± SD (range)

## Discussion

In the present study, we compared the patient-reported outcome among three groups with different lateral laxities. The important finding of this study was that excessive lateral laxity possibly resulted in unfavourable patient-reported outcome. Moreover, slight lateral laxity of 2–5 mm might have no effect on patient-reported outcome, and this slight varus imbalance could be acceptable. However, lateral laxity of more than 5 mm decreased the symptom score, and this may be attributable to the fact that the excessive lateral laxity is not physiologically comparable to that of the normal knee.

Soft tissue balance other than alignment that can be manipulated and achieved during the operation ensures good clinical outcomes. Romero et al. reported that increased lateral laxity (mean 11.0°) was more symptomatic than the control mean of 7.0° after CR TKA using stress X-ray evaluation 1 year after TKA [6]. However, there have been few studies evaluating lateral laxity during surgery and patient-reported outcome.

The balancing of TKA has been considered to be rectangular and equal, both in flexion and extension. To achieve the rectangular gap, medial release must occur, e.g. the MCL and semimembranosus tendon have to be released. This might lead to medial instability, which is reported to be associated with poor functional outcome [7–10]. However, it is important to obtain a degree of lateral laxity to achieve good clinical outcomes. Moreover, evaluation of patient-reported outcome including satisfaction has become important after TKA.

Factors affecting patient satisfaction have been reported to be postoperative alignment and ROM [4, 14]. In this study, there were no significant differences among the three groups in terms of patient satisfaction. However, the satisfaction scores tended to decrease with an increase in lateral laxity to more than 5 mm. Moreover, there were no significant differences among groups in terms of postoperative ROM. Matsuda et al. reported that the balanced group, in which the difference between varus and valgus in knee extension was less than 2 mm, showed improved ROM compared to that in the unbalanced group after mobile-bearing TKA (40 PS TKA and 40 CR TKA) [15]. Conversely, it was reported that lateral laxity at 90° of flexion had a positive effect on the postoperative ROM after CR TKA [16, 17]. PS TKA resulted in a significantly larger joint gap in flexion than did the CR TKA [18, 19]. In the present study, we examined the laxity after PS TKA. NexGen LPS flex was designed the post engage to the cam from the 75° flexion angle. The femoral component began rollback from the 90° flexion angle. However, the relationship between lateral laxity and flexion angle is still unclear and further study is needed to clarify this relationship.

Our study has some limitations. First, we did not assess postoperative rotational axis of the implanted femoral component. Femoral rotation should affect the patellofemoral pressure and clinical outcome. However, in this study, we used a measured resection technique and the rotation of the femur was usually determined at 3° or 5° relative to the posterior condylar axis. Second, this study involved a small sample size; thus, studies with greater number of patients are needed to further validate our findings in the future. Third, we evaluated only PS fixed TKA approach with the multi-radius design. In the future, evaluations using variable prosthesis designs are needed.

## Conclusion

Although slight lateral laxity of 2–5 mm could have no effect on patient-reported outcome after posterior-stabilised fixed TKA, lateral laxity of more than 5 mm deteriorated the patient-reported outcome. This finding could lead to avoidance of excessive medial release in soft tissue balancing in TKA.

## Data Availability

The datasets generated during and/or analysed during the current study are not publicly available due to patient confidentiality but are available from the corresponding author on reasonable request.

## Abbreviations

TKA: Total knee arthroplasty
PS: Posterior-stabilised
CR: Cruciate retaining
MCL: Medial collateral ligament
